# mTOR signaling pathway and mTOR inhibitors in cancer: progress and challenges

**DOI:** 10.1186/s13578-020-00396-1

**Published:** 2020-03-10

**Authors:** Zhilin Zou, Tao Tao, Hongmei Li, Xiao Zhu

**Affiliations:** 1 Guangdong Key Laboratory for Research and Development of Natural Drugs, Guangdong Medical University, Zhanjiang, China; 2 Marine Medical Research Institute of Guangdong Zhanjiang (GDZJMMRI), Southern Marine Science and Engineering Guangdong Laboratory Zhanjiang, Guangdong Medical University, Zhanjiang, China; 3 Department of Pathology, Guangdong Medical University, Dongguan, China; 4 Department of Gastroenterology, Zibo Central Hospital, Zibo, China

**Keywords:** mTOR signaling pathway, mTOR inhibitor, Tumor metabolism, Autophagy, Apoptosis

## Abstract

Mammalian target of rapamycin (mTOR) regulates cell proliferation, autophagy, and apoptosis by participating in multiple signaling pathways in the body. Studies have shown that the mTOR signaling pathway is also associated with cancer, arthritis, insulin resistance, osteoporosis, and other diseases. The mTOR signaling pathway, which is often activated in tumors, not only regulates gene transcription and protein synthesis to regulate cell proliferation and immune cell differentiation but also plays an important role in tumor metabolism. Therefore, the mTOR signaling pathway is a hot target in anti-tumor therapy research. In recent years, a variety of newly discovered mTOR inhibitors have entered clinical studies, and a variety of drugs have been proven to have high activity in combination with mTOR inhibitors. The purpose of this review is to introduce the role of mTOR signaling pathway on apoptosis, autophagy, growth, and metabolism of tumor cells, and to introduce the research progress of mTOR inhibitors in the tumor field.

## Background

The mTOR forms two structurally and functionally distinct complexes called the mammalian target of rapamycin complex 1 (mTORC1) and mammalian target of rapamycin complex 2 (mTORC2). mTORC1 is comprised of mTOR, raptor, GβL and deptor, while mTORC2 is composed of mTOR, Rictor, GβL, PRR5, deptor, and SIN1. mTORC1 integrates signals from multiple growth factors, nutrients, and energy supply to promote cell growth when energy is sufficient and catabolism when the body is hungry. mTORC1 mainly regulates cell growth and metabolism, while mTORC2 mainly controls cell proliferation and survival [1]. Studies have shown that mTOR is involved in many signaling pathways in the body, including phosphoinositide-3-kinase (PI3K)/AKT, tuberous sclerosis complex subunit 1 (TSC1)/tuberous sclerosis complex subunit 2 (TSC2)/Rheb, LKBL/adenosine 5′-monophosphate-activated protein kinase (AMPK), VAM6/Rag GTPases and so on [2]. It influences transcription and protein synthesis by integrating various signal stimulation, and finally regulates apoptosis, growth, and autophagy of cells [3]. Scientists have also linked mTOR to various disease processes, such as tumor formation, arthritis, insulin resistance and osteoporosis [4, 5]. Among them, mTOR plays a key role in tumor tumorigenesis and development. And multiple studies have suggested that tumors typically over-activate the AKT/mTOR signaling pathway [6, 7]. Therefore, mTOR inhibitors are widely used in the research of targeted therapy for tumors, organ transplantation, rheumatoid arthritis, and other diseases. The purpose of this review is to elucidate the relationship between mTOR signaling pathway and tumor development and to introduce the research progress of mTOR inhibitors.

## The relationship between mTOR signaling pathway and tumors

### Tumor growth and proliferation

Under normal circumstances, mTOR is a major regulator of cell growth and division. However, in tumor cells, abnormally activated mTOR sends signals that encourage tumor cells to grow, metastasize, and invade new healthy tissues [8]. Among them, PI3K/phosphate and fungi homology deleted on chromosome 10 (PTEN)/AKT/TSC pathway is the main activator of mTORC1, and gene mutations in this pathway can lead to malignant tumors [9]. In addition, the expression of PTEN is often eliminated by epigenetic, genetic, and post-transcriptional modification to up-regulate the PI3K/Akt/mTOR pathway in most malignant tumors [10].

Hou et al*.* [11] found that mutations in the PTEN gene led to abnormal activation of the PI3K/PTEN pathway in hepatic cell carcinoma (HCC). Furthermore, deletion of the PTEN gene induces the expression of B7-H1, which leads to immunosuppression and increases tumor progression and invasion [12]. In liver cancer, the PI3K/PTEN/Akt/mTOR pathway activated is involved in tumor invasion and metastasis by up-regulating matrix metallopeptidase 9 (MMP-9) [13]. Similarly, the PI3K/Akt/mTOR signaling pathway has been found to control the proliferation and survival of colon cancer stem cells (CCSC). In sporadic colon cancer, CCSC may cause recurrence and metastasis [14]. Xie et al*.* [15] found that liver kinase B1 (LKB1) gene mutation or extracellular growth signal could activate mTORC1. MTORC1 inhibits the activity of ring finger protein 168 (RNF168) protein and promotes its degradation by phosphorylating the 60th serine of RNF168. This will significantly reduce the ubiquitination modification of histone H2A and H2A histone family member X (H2AX) after DNA damage, which will inhibit the response to DNA damage and reduce the stability of the genome, leading to the promotion of malignant cell transformation and cancer. In addition, existing research shows that Rheb is a GTPase that binds and activates mTORC1 when GTP is loaded. Deng et al*.* [16] reported that the ubiquitination of Rheb was regulated by growth factor signals. Ubiquitinated Rheb inhibits Rheb activity by promoting Rheb binding to TSC2, leading to the inhibition of mTORC1 expression. In addition to the mTORC1 pathway, the mTORC2 pathway is also involved in the regulation of the occurrence and development of tumor cells. Wang et al*.* [17] demonstrated that OTU deubiquitinase 7B (OTUD7B) reduced ubiquitination level of GβL to prevent GβL from interacting with SIN1, leading to activation of mTORC2/AKT signaling pathway and down-regulation of mTORC1 expression. This partially activates AKT oncogenic signaling and promotes tumorigenesis. However, the ubiquitin ligase TNF Receptor Associated Factor 2 (TRAF2) has the opposite effect by increasing the level of GβL ubiquitination. Similarly, Kovalski et al*.* [18] proved that Ras mutations can bind to mTOR of mTORC2 and mitogen-activated protein kinase-associated protein 1 (MAPKAP1) to promote the activity of mTORC2 kinase, thus initiating downstream proliferative cell cycle transcription programs.

**Fig. 1:**
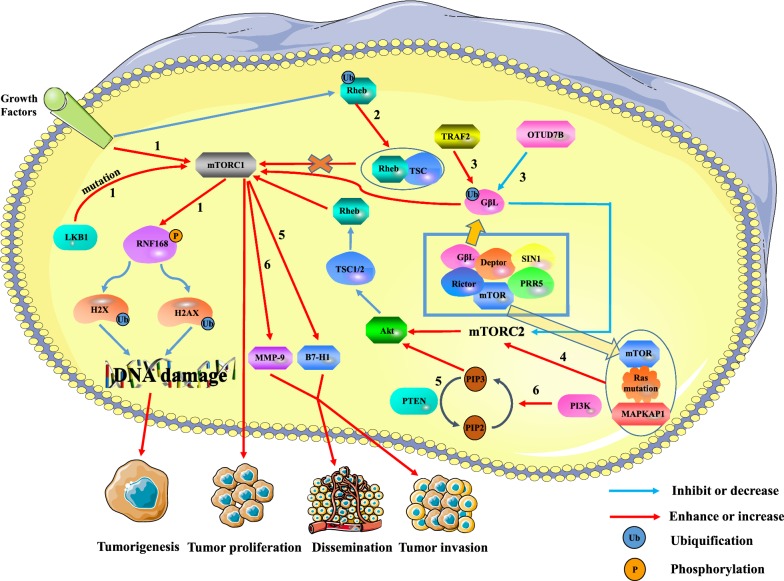
The relationship between mTOR and tumors. Overactivation of mTORC1 can promote tumor formation, proliferation, and metastasis, while mTORC2 can regulate the expression of mTORC1 through the mTORC2/AKT/TSC/Rehb pathway. Pathway 1: The extracellular growth signals and intracellular LKB1 mutations activate mTORC1, which reduces the ubiquitination of histone H2A and H2A after DNA damage by phosphorylating RNF168. This can lead to damage to DNA repair and promote the formation of tumors. Pathway 2: The ubiquitination of Rheb reduces Rheb activity by promoting Rheb binding to TSC2. The down-regulation of Rehb reduces the activation of mTORC1, leading to the inhibition of tumor growth. Pathway 3: TRAF2 and Otud7B respectively regulate mTORC1/2 activity by up-regulating or down-regulating the ubiquitination level of G beta L of mTORC2. TRAF2 enhanced the activity of mTORC1 and inhibited the activity of mTORC2. Although down-regulation of mTORC2 expression inactivates the AKT/TSC/Rehb/mTORC1 pathway, overall mTORC1 activity is enhanced. However, Otud7B has the opposite effect on TRAF2. Pathway 4: Mutated Ras binds mTOR and MAPKAP1 of mTORC2 to promote mTORC2 expression. The up-regulation of mTORC2 promotes tumor proliferation through the AKT/TSC/Rehb/mTORC1 pathway. Pathway 5: Deletion of the PTEN gene induces the expression of B7-H1 to increase tumor progression and invasion. Pathway 6: The PI3K/PTEN/AKT/mTOR pathway is involved in the invasion and metastasis of liver cancer by up-regulating MMP-9

In summary, mTOR is always stimulated in tumors to maintain the growth, survival and proliferation of tumor cells, and plays a key role in tumor cell biology (Fig. 1).

### Tumor metabolism

mTOR is activated when nutrients are sufficient, which promotes anabolism and energy storage and utilization. When nutrients are relatively scarce, the body must inhibit the activation of mTOR to keep cell material and energy stable. Tumor cells require large amounts of proteins, lipids, and nucleotides to respond to their needs for growth and division [19]. Therefore, abnormal activity of the mTOR pathway often occurs in tumors, because mTOR plays a core role in regulating metabolism.

In breast cancer cells, the PI3K/AKT/mTORC1/sterol regulatory element-binding protein (SREBP) pathway is the main mechanism to induce new lipid synthesis and promote tumor proliferation [20]. Pyruvate kinase (PK) is involved in sugar metabolism while fatty acid synthase (FASN) is involved in the synthesis of fatty acid (FA). Tao et al*.* [21] found that down-regulating the expression of pyruvate kinase M2 (PKM2) deactivates the AKT/mTOR signaling pathway, thereby reducing the expression of SREBP-1c. The reduced expression level of SREBP-1c inhibits the generation of FA by inhibiting the transcription of the FASN gene, resulting in the inhibited growth of tumor cells. In addition, Di Malta et al*.* [22] reported that the up-regulated transcription factor enhancer (TFE) gene can activate the Rag GTPase/mTORC1 pathway. In normal cells, this pathway is activated so that cells can better absorb nutrients to maintain physiological functions. In tumor cells, this pathway is often over-activated to meet the nutritional needs of the rapidly growing tumor cells. However, Guri et al*.* [23] explored that mTORC2 promoted the production of sphingomyelin and cardiolipin in HCC. On the one hand, sphingomyelin and cardiac phospholipids are both structural components of cell biofilms. On the other hand, the metabolism and transport of cardiac phospholipids contribute to the proper functioning of mitochondria, so they must be supplied in large quantities in rapidly proliferating tumor cells [24]. These results suggest that the mTORC2 signaling pathway promotes HCC proliferation and energy-related lipid production.

As mentioned above, the metabolisms of tumor cells can be regulated by the mTOR pathway to meet their proliferative and nutritional needs. Conversely, tumor cell metabolism can also promote tumor growth through the mTOR pathway. Ericksen et al*.* [25] demonstrated that the reduction of branched-chain amino acid (BCAA) decomposition could promote the occurrence and development of tumors by enhancing the activity of mTORC1. They also concluded that the activity of the key enzyme in the BCAA catabolism process was highly correlated with tumor invasion. Therefore, BCAAs accumulation caused by inhibition of BCAA catabolism in liver tumor tissues may be the primary mechanism of chronic activation of tumor mTORC1. Similarly, Shi et al*.* [26] showed that the expression of adenosine A2a receptor (A2aR) in gastric cancer (GC) tissues was increased, and the expression of A2aR was positively correlated with the GC stage. The results suggest that adenosine activates the PI3K/AKT/mTOR signaling pathway by binding to A2aR, which ultimately promotes the progress of GC. Madak-Erdogan et al*.* [27] found that free fatty acids (FFAs) activated estrogen receptor α (ERα) and mTOR pathways, which were correlated with higher proliferation and invasiveness of ER (+) breast cancer cells.

**Fig. 2:**
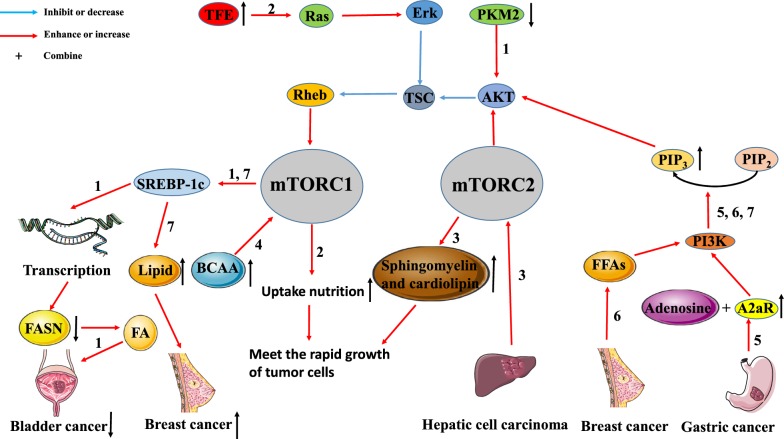
Interaction between tumor metabolism and the mTOR signaling pathway. The mTOR pathway is closely related to tumor metabolism. Pathway 1: In bladder cancer, down-regulation of PKM2 expression reduces SREBP-1 expression through inactivated AKT/TSC/Rehb/mTORC1 pathway. The down-regulation of SREBP-1c inhibits FA generation by inhibiting FASN transcription, leading to the inhibition of tumor growth.). Pathway 2: Up-regulation of TEF in tumors activates the Ras/Erk/TSC/Rehb/mTORC1 pathway. Activation of this pathway will promote the uptake of nutrients by tumor cells to meet the needs of the rapid growth of tumors. Pathway 3: HCC can increase sphingomyelin and cardiolipin production by activating mTORC2. Large amounts of sphingomyelin and cardiolipin are used to assemble cell membranes, which also meet the needs of rapid tumor proliferation. Pathway 4: The accumulation of BCAA can promote the occurrence and development of tumors by activating mTORC1. Pathway 5: A2aR, which is highly expressed in gastric cancer, binds adenosine to activate the PI3K/AKT/mTORC1 pathway. Pathway 6: In breast cancer, FFAs promotes tumor proliferation and metastasis by activating the PI3K/AKT/mTORC1 pathway. Pathway 7: The PI3K/AKT/mTORC1/SREBP pathway promotes breast cancer proliferation by inducing new lipid synthesis

These studies indicate that the mTOR signaling pathway is closely related to tumor metabolism, and provide theoretical support for the combined application of mTOR inhibitors and some drugs that interfere with tumor metabolism (Fig. 2).

### Immune cells

Tumors can develop immune tolerance by suppressing the immune system's ability to recognize and kill tumor cells. Tumor immunotherapy is a hot research topic in recent years [28], and a variety of evidence shows that mTOR pathway, which is often abnormally activated in tumors, can regulate the differentiation and function of immune cells.

#### T cells

T-progenitor cells from the bone marrow enter the thymus, where they differentiate into two types of cells: αβ T cells and γδ T cells. Through the gene knockout experiment, Yang et al*.* [29] proved that RAPTOR deletion in mTORC1 would break the remodeling process of oxidative metabolism and glucose metabolism during T cell differentiation. This triggers the production of reactive oxygen species (ROS), which disrupts the differentiation of αβ T cells and increase the differentiation of γδ T cells. In addition, Essig et al*.* [30] showed that roquin could down-regulate the expression of the PI3K/mTOR pathway. The downregulation of the PI3K/mTOR pathway not only inhibits the differentiation and activation of traditional T cells, but also limits the specialization of Treg cells. Pollizzi et al*.* [31] found in mouse models that activation of mTORC1 promoted the production of a cluster of differentiation (CD) 8+ effector T cells with high glycolysis. These T cells retain the effector phenotype but do not convert to a memory phenotype. In contrast, T cells with reduced activity of mTORC1 have the characteristics of memory cells but cannot differentiate into effector cells, and they cannot produce an immune memory response due to the defective metabolic function. Further studies showed that mTORC1 could affect the effector function of CD8+ T cells, while mTORC2 activity regulated the memory ability of CD8+ T cells.

In general, the mTOR signaling pathway can affect the differentiation and function of T cells. These studies extend our current understanding of T cell biology.

#### Natural killer cells and dendritic cells

The mTOR signaling pathway not only regulates T cells, but is closely related to the differentiation and functions of dendritic cells (DCs) and natural killer (NK) cells. DC has a strong antigen presentation ability, and NK cells are important immune cells in the body. Wang et al*.* [32] proved that mTORC1 and mTORC2 regulate NK cell effects in a unique way. They found that mTORC2 negatively regulates NK cell function mainly by inhibiting the signal transducer and activator of transcription 5 (STAT5)/solute carrier family 7 member 5 (SLC7A5) axis. While mTORC1 positively regulates the activity of mTORC2 by maintaining the CD122-mediated interleukin (IL)-15 signaling pathway. In addition to NK cells, DC is also believed to be related to the mTOR signaling pathway in recent years. Chen et al*.* [33] concluded that apoptosis of DC-derived from bone marrow mononuclear (BMM) cells was reduced after treatment with mTOR inhibitors. They also found that BMM cell-derived DCs had better antigen presentation capabilities and that e7-specific cytotoxic CD8+ T lymphocytes activated by these DCs had greater anti-tumor activity. Therefore, mTOR inhibitors can enhance the efficacy of tumor immunotherapy by extending the life span of DC, improving antigen presentation and antigen processing ability. These studies provide ideas for targeting NK and DC cells for anti-tumor therapy.

#### Macrophages

M1-type macrophages can kill tumor cells in multiple ways, while M2-type macrophages promote the occurrence, invasion, and metastasis of the tumors. Zhihua et al*.* [34] reported that the expression of microRNA (miRNA)-30c was significantly reduced in GC. Further studies showed that hypoxia-inducible factor-1α (HIF-1α) inhibited miRNA-30c expression. The downregulation of miRNA-30c will reduce mTOR activity and glycolysis in tumor-related macrophages. This will eventually promote GC growth and metastasis by inhibiting the differentiation and function of M1-type macrophages.

In general, the mTOR pathway, which is often activated in tumors, promotes tumor growth by regulating the differentiation and function of immune cells. This will play a positive role in the exploration of new immunotherapy and the improvement of tumor treatment.

### Tumor drug resistance

Targeting tumor cell molecular pathways is the way we treat various cancers, but tumors inevitably develop drug resistance [35]. Once the tumor becomes resistant, the side effects of the treatment increase while the effectiveness of the drug decreases significantly.

#### The tumors of digestive system

Studies have demonstrated that mitochondrial uncoupling protein 2 (UCP2) leads to tumor resistance to multiple anticancer drugs by reducing ROS generated by mitochondrial metabolism. Dando et al*.* [36] proved that the combination of genipin and everolimus could synergistically inhibit the growth of pancreatic adenocarcinoma (PAAD) cells and induce autophagy of tumor cells. This is because inhibition of UCP2 in PADD cells activates the Akt/mTOR pathway by a ROS dependent mechanism, which reduces the anti-proliferation effect of UCP2 inhibitor genipin. The hedgehog pathway mediated by zinc finger protein GLI1 plays a major role in GC. However, Yao et al*.* [37] demonstrated that the AKT/mTOR pathway can activate GLI1. Moreover, the GLI1 and p-AKT expressions were correlated with tumor cell metastasis and drug resistance, and the expression level was negatively correlated with the prognosis of patients with GC.

#### Respiratory tumors

The wee1 inhibitor AZD1775, which targets DNA repair and cell cycle checkpoints, has been shown to be effective in some lung cancer patients, but drug resistance is also common [38, 39]. In this regard, Sen et al*.* [40] found that AXL receptor tyrosine kinase (ARK) expression in AZD1775-resistant small cell lung cancer (SCLC) was up-regulated. ARK can directly or through mTOR activate the extracellular regulated protein kinases (ERK) pathway to recruit and activate checkpoint kinase 1 (CHK1). These results suggest that ARK can enhance DNA damage repair by activating CHK1, which ultimately invalidates the Wee1 inhibitor. Similarly, Ye et al*.* [41] demonstrated that in non-small cell lung cancer (NSCLC), transmembrane-4 L-six family member-1 (TM4SF1) regulates tumor sensitivity to chemotherapy drugs by regulating the expression of discoid domain receptor 1 (DDR1)/Akt/ERK/mTOR pathway.

#### Kidney cancer and skin cancer

Tyrosine kinase inhibitors (TKI) inhibitors can be used to treat renal cell carcinoma (RCC), but some RCC patients will develop drug resistance [42]. Ishibashi et al*.* [43] reported that the combined treatment of interleukin-6 receptor (IL-6R) inhibitor and low-dose TKI inhibitor was more effective in inhibiting RCC growth and angiogenesis in vivo compared with the use of TKI inhibitor alone. This is because low doses of TKI inhibitors induce high levels of IL-6, which activates the AKT/mTOR pathway. These results suggest that the mechanism of failure of TKI inhibitors in some RCC patients is related to elevated IL-6 activation of the mTOR signaling pathway.

B-Raf proto-oncogene (BRAF) mutations occur in nonmelanoma skin cancer (NMSC), but BRAF inhibitors have not been used in such tumors. The main reason is that BRAF mutated NMSC has primary or secondary resistance to BRAF inhibitors. Sen et al*.* [44] found a strong correlation between PI3K/mTOR signaling pathway and BRAF inhibitor resistance. This suggests that activation of the mTOR pathway may lead to BRAF mutated NMSC resistance to BRAF inhibitors. In addition, Obenauf et al*.* [45] found that BRAF, anaplastic receptor tyrosine kinase (ALK) and epidermal growth factor receptor (EGFR) inhibitors induced some secretion signals in cancer cells, which led to the proliferation and metastasis of drug-resistant tumor cells. Further studies have found that in melanoma cells treated with BRAF inhibitor, the downregulation of transcription factor FOS related antigen-1 (FRA1) activates multiple signaling pathways, among which the PI3K/AKT/mTOR pathway plays a major role. When the BRAF and PI3K/AKT/mTOR signaling pathways are simultaneously inhibited, the growth of drug-resistant BRAF mutated human melanoma cells can be inhibited.

The above studies suggest that tumor cells can evade anti-tumor drug-induced cell death by activating the intracellular mTOR signaling pathway, so the activation of the mTOR signaling pathway may be one of the mechanisms of drug resistance in tumors. Drug combinations targeting the mTOR signaling pathway may be used to treat tumors that have developed resistance.

### Autophagy and apoptosis of cancer

On the one hand, autophagy can keep the genome stable by removing damaged organelles and misfolded proteins, so it can inhibit the growth of cancerous cells [46]. On the other hand, autophagy provides the tumor with more nutrients, which strengthens the tumor's ability to cope with extreme environments [47, 48]. In addition, the unlimited proliferation of tumors is partly due to the inhibition of tumor cell apoptosis.

Sun et al*.* [49] concluded that mTOR inhibits the expression of glycogen synthase kinase-3 (GSK-3) in prostate cancer cells. The down-regulation of GSK-3 will inhibit the caspase-3 signaling pathway, leading to the reduction of ROS production. Decreased ROS inhibits apoptosis of tumor cells to protect prostate cancer cells. However, Zou et al*.* [50] reported that mTORC2 prevented cancerous inhibitor of protein phosphatase 2A (CIP2A) from binding to protein phosphatase 2A (PP2A) to restore PP2A activity. PP2A reduces the transcription of miR-9-3p and upregulates the expression of E2F transcription factor 1 (E2F1) by promoting the degradation of c-Myc, so it inhibits the apoptosis of tumor cells. In addition, Yang et al*.* [51] reported that the expression of p-AKT, p-mTOR, P62 and B-cell lymphoma-2 (BCL-2) was significantly decreased in oral squamous cell carcinoma (OSCC) cells with long non-coding RNA (lncRNA) CASC9 knockdown, while the expression of BCL2 associated X (BAX) was increased. These results suggest that lncRNA CASC9 inhibits autophagy-mediated apoptosis through the AKT/mTOR pathway, which promotes OSCC cell proliferation.

In summary, the above studies indicate that mTOR signaling pathway can promote the occurrence and progression of tumors by regulating autophagy and apoptosis of tumor cells. In addition, the above research also provides theoretical support for clinical research on anticancer targeted drugs, which is of great significance.

## The research progress of mTOR inhibitors in tumors

### New mTOR inhibitors

Because the mTOR signaling pathway is active in most human cancers, scientists have developed first and second-generation mTOR inhibitors for cancer treatment. The U.S. food and drug administration approved temsirolimus and everolimus for use in kidney or breast cancer. Temsirolimus is used to treat advanced RCC, while everolimus is used to treat RCC that has failed to respond to sunitinib or sorafenib, in combination with exemestane for breast cancer with hormone receptor (+) and Her2 (−), pancreatic neuromedicinal tumors (pNET) and subependymal giants-cell astrocytoma (SEGA).

Rodrik-Outmezguine et al*.* [52] connected the first and second-generation of mTOR inhibitors together to form the third generation of mTOR inhibitor rapalink-1, which can simultaneously target two targets on the mTOR enzyme. Rapalink-1, with its potent anticancer activity, reduces the size of tumors resistant to first- or second-generation mTOR inhibitors. This method provides a new idea and model for the design of new anticancer, antiviral and antibacterial drugs. In addition to common clinical mTOR inhibitors, a variety of drugs have been found to inhibit mTOR activity. Nguyen et al*.* [53] explored that the Halitulin analog ICSN3250 is a specific mTOR inhibitor, which can compete and replace phospholipids acid in the FRB domain of mTOR. Previous studies have shown that the newly discovered drug LY3023414 inhibits the PI3K/mTOR/DNA dependent protein kinase (DNA-PK) pathway in vitro tumor cells and has anti-tumor proliferation activity. Bendell et al. [54] conducted clinical trials and concluded that LY3023414 achieved sustained partial remission in endometrial cancer patients with phosphoinositide-3-kinase regulatory subunit 1 (PIK3R1) and phosphate and PTEN mutations. In addition, Plews et al. [55] proved that OSU-53 could inhibit the growth of thyroid cancer cells in vitro by activating AMPK to inhibit the mTOR pathway or directly inhibit the mTOR pathway.

These results suggest that a variety of newly discovered mTOR inhibitors are effective in many tumors. In the future, these drugs can be perfected for clinical research, so that they can be used for clinical treatment.

### New progress in mTOR inhibitors

With the development of the mTOR pathway, many scientists have studied the effects of mTOR inhibitors in different tumors [48, 56–58]. Because conventional platinum chemotherapy failed to respond to ovarian clear cell carcinoma (OCCC), Caumanns et al. [59] conducted drug testing of mtorc1/2 inhibitor AZ D8055 in the OCCC cell lines. The results showed that the OCCC cell line was sensitive to AZD8055, and AZD8055 was validated in a xenotransplantation model. Similarly, Hanna et al. [60] demonstrated that everolimus was effective in undifferentiated thyroid cancer patients with PI3K/mTOR/Akt mutations. Morran et al. [61] reported that rapamycin effectively blocked the proliferation and development of pancreatic cancer cells in mice with Kirsten rat sarcoma viral oncogene (KRAS) activation and PTEN mutation. Moreover, pancreatic cancer with Kirsten rat sarcoma viral oncogene (KRAS) activation and p53 mutation did not respond to rapamycin. The mTOR inhibitor temsirolimus can down-regulate the expression of tumor hypoxia-inducing factor and block the cell cycle in G1 phase to inhibit the growth of cancer cells and tumor angiogenesis. Korfel et al. [62] explored that temsirolimus can effectively treat primary central nervous system lymphoma (PCNSL), but the response was relatively short. In addition to the above effects, scientists also found that the mTOR inhibitor has a protective effect. Functional pNET can secrete 5-hydroxytryptamine (5-HT) into the blood, and over time high 5-HT in the blood can damage the heart. Orr-Asman et al. [63] found that mTOR kinase inhibitor CC-223 reduced myocardial damage by reducing cardiac valve fibrosis compared with pNET mice treated with either placebo or rapalog alone. However, Goldman et al*.* [64] reported that mTOR inhibitors could reduce infertility caused by ovarian damage due to chemotherapy. Table 1 summarizes the research progress of mTOR inhibitors.

**Table 1:** Summary of the research phase of the mTOR inhibitors

mTOR inhibitors	Applied tumor	Phase	References
Everolimus	RCC	FDA approved	–
Temsirolimus	Advanced RCC	FDA approved	–
ICSN3250	Colon cancer cell	Pre-clinical studies	Nguyen et al. [53]
LY3023414	Solid tumor or lymphoma	Phase I clinical trial	Bendell et al. [54]
OSU-53	Thyroid cancer cell	Pre-clinical studies	Plews et al. [55]
AZD8055	OCCC cell	Pre-clinical studies	Caumanns et al. [59]
Everolimus	Aggressive and RAIR thyroid cancer	Phase II clinical trial	Hanna et al. [60]
Rapamycin	Pancreatic cancer	Pre-clinical studies	Morran et al. [61]
Temsirolimus	PCNSL	Phase II clinical trial	Korfel et al. [62]

*RCC* renal cell carcinoma, *OCCC* ovarian clear cell carcinoma, *RAIR* radioactive iodine-refractory, *PCNSL* primary central nervous system lymphoma, *FDA* Food and Drug Administration

### TSC mutations and the effects of mTOR inhibitors

TSC1/2 is upstream of mTOR and regulates mTOR activity through Rheb. Some studies have shown that in some tumors, patients with TSC mutations are unusually sensitive to mTOR inhibitors. Wang et al*.* [65] found in a case of renal angiomyolipoma that somatic cell mutations in the patient stopped the translation of TSC2, which led to the activation of mTORC1, and the application of everolimus effectively reduced tumor growth and distal metastasis. Similarly, TSC mutations in RCC, chromophobe RCC, and metastatic RCC patients were reported unusually sensitive to mTOR inhibitors [66–69]. Furthermore, metastatic RCC patients with TSC mutation benefit from rapalogs are more common than the advanced patients [70]. In addition to renal tumors, hepatitis B virus (HBV)-associated HCC with TSC mutations had a similar effect [71]. Levine et al*.* [72] analyzed data from patients with endometrioid adenocarcinoma and found that patients with TSC2 mutations had longer progression-free survival than patients with other drugs. This phenomenon may be due to the abnormal activation of mTORC1 by the TSC mutation, which makes such somatic mutant patients unusually sensitive to mTOR inhibitors. Therefore, the TCS/mTOR pathway gene sequencing in tumor patients can be used to guide clinical drug therapy.

### Combination therapy with mTOR inhibitors

There are feedback mechanisms in the PI3K/AKT signaling pathway, including positive and negative feedback. In the negative feedback mechanism, when the PI3K/AKT/mTORC1 pathway is activated, the downstream S6K1 is activated. Activated S6K1 phosphorylates downstream insulin receptor substrate (IRS) so that the IRS cannot activate PI3K. This reduces the phosphorylation of phosphatidylinositol (4,5)-bisphosphate (PIP2) to phosphatidylinositol (3,4,5)-trisphosphate (PIP3), which ultimately inhibits AKT activity. In the positive feedback mechanism, activation of the PI3K/AKT pathway also activates the downstream inhibitor of nuclear factor kappa-B kinase (IKK)/nuclear factor kappa-B (NF-κB) pathway. Activated NF-B inhibits PTEN expression to reduce the dephosphorylation of PIP3, which leads to the accumulation of PIP3 and continued activation of AKT. When mTOR inhibitor inhibits mTORC1, it will inhibit the inhibitory effect of S6K1 on IRS, thus enhancing the activation of the PI3K/AKT signaling pathway [73]. Thus, although the mTOR pathway is involved in almost every process of cancer, including cell growth, proliferation, metabolism, and immune response, the activation of AKT by mTOR inhibitors limits its clinical effectiveness.

However, many studies have shown that other antitumor drugs combined with mTOR inhibitors can overcome this resistance [74]. Motzer et al. [75] demonstrated that the combination therapy of Vascular endothelial growth factor (VEGF) inhibitor lenvatinib and everolimus had good efficacy in advanced or metastatic RCC. In head and neck squamous cell carcinoma (HNSCC), MAPK/ERK kinase (MEK)/ERK/activator protein 1 (AP-1), NF-κB and mTOR signaling pathways are frequently activated. Mohan et al. [76] showed that PI3K/mTOR inhibitor PF-384 could not completely inhibit the growth of xenograft HNSCC. While the combined use of PF-384 and the MEK inhibitor PD-901 can inhibit the production of IL-8 and VEGF and the reverse activation of downstream NF-κB and AP-1, thus significantly inhibiting tumor growth, proliferation, anti-apoptosis, and angiogenesis. Zhang et al. [77] concluded that inhibition of androgen receptor (AR) in HCC could feedback to activate the AKT/mTOR signaling pathway, while mTOR reduced AR degradation and promoted the expression of nuclear AR protein. In addition, Chen et al. [78] found in breast cancer that mTOR inhibitor AZD8055 can prevent the heat shock protein (HSP) 90 inhibitor AUY922 from up-regulating the response of HSP70 and HSP27 in breast cancer, while HSP90 inhibitor can block the activation of PI3K/Akt caused by mTOR inhibitor. Therefore, mTOR inhibitors can synergistically inhibit tumor growth in combination with HSP90 inhibitors or AR inhibitors.

mTOR inhibitor combination therapy has also shown activity in many types of lung cancer. Christopoulos et al. [79] reported that carboplatin and paclitaxel combined with everolimus were effective and well-tolerated in patients with metastatic large-cell neuroendocrine carcinoma (LCNEC). In addition, Hai et al. [80] showed that the combined use of mTOR and Wee1 inhibitors could synergically inhibit KRAS mutated NSCLC cell lines. This is because mTOR inhibitors inhibit DNA repair by reducing cyclin D1, which enhances the inhibition effect of Wee1 by enhancing DNA damage in NSCLC cells.

These studies suggest that a combination of mTOR inhibitors may enhance the activity of a variety of antitumor drugs, compared with drugs taken alone (Table 2). These studies guide the clinical use of mTOR inhibitors and other anti-tumor drugs.

**Table 2:** Summary of mTOR inhibitors in combination with other antitumor drugs

mTOR inhibitors	Combined drug	Applied tumor	The effect	References
Everolimus	VEGF inhibitor lenvatinib	RCC	Progression-free survival is significantly extended compared to using them separately	Motzer et al. [75]
PF-384	MEK inhibitor PD-901	HNSCC	They inhibit the production of IL-8 and VEGF and the activation of NF-κB and AP-1	Mohan et al. [76]
AZD8055	HSP90 inhibitor AUY922	Breast cancer	AZD8055 inhibits the upregulation of HSP70 and HSP27 induced by AUY922, while AUY922 blocks the activation of PI3K/Akt induced by AZD805	Chen et al. [78]
Rapamycin	AR inhibitor enzalutamide	HCC	Rapamycin inhibits the AKT/mTOR signaling pathway activated by enzalutamide, while enzalutamide inhibits the up-regulation of AR expression caused by rapamycin	Zhang et al. [77]
Everolimus	Carboplatin and paclitaxel	LCNEC	They improve the overall response rate and disease control rate	Christopoulos et al. [79]
AZD2014	Wee1 inhibitor AZD1775	NSCLC	AZD2014 enhances the effect of AZD1775 by reducing cyclin D1 to enhance DNA damage	Hai et al. [80]

*AP-1* activator protein 1, *AR* androgen receptor, *HCC* hepatic cell carcinoma, *HNSCC* head and neck squamous cell carcinoma, *HSP90* heat shock protein 90, *IL-8* interleukin-8, *LCNEC* large-cell neuroendocrine carcinoma, *MEK* MAPK/ERK kinase, *NSCLC* non-small cell lung cancer, *RCC* renal cell carcinoma, *VEGF* vascular growth factor

## Conclusion and prospect

The mTOR signaling pathway is closely related to tumors, and it is closely related to its cell growth, metabolism, apoptosis and autophagy [47, 81]. For example, the mTOR signaling pathway can affect gene transcription and protein synthesis to regulate cell growth and proliferation, affect the immune cell differentiation to participate in immune regulation, and play an important role in tumor metabolism. A number of studies have shown that a variety of new mTOR inhibitors show high anti-tumor activity in clinical studies, and the use of mTOR inhibitors in combination with other anti-tumor drugs has a significant effect. This review describes the relationship between the mTOR signaling pathway and tumor cell growth, metabolism, apoptosis and autophagy). This review also introduces the role of mTOR signaling in tumors of various organs and the research progress on the application of mTOR inhibitors in tumors, which indicates the importance of the mTOR signaling pathway in the tumor fields.

However, the role of the mTOR signaling pathway has not been clearly studied, and although mTOR inhibitors can inhibit tumor cell growth, their ability to induce tumor cell death is limited. The mechanisms of tumors are complex and involve many signaling pathways. Inhibition of certain signaling pathways may lead to feedback activation of other signaling pathways, so although mTOR inhibitor combination therapy is more effective, its effect is limited. In addition, clinical trials have demonstrated that the side effects of treatment with mTOR inhibitors cannot be ignored. It is hoped that the action mechanisms of the mTOR signaling pathway can be clearly studied in the future, and then selective mTOR inhibitors can be developed to improve anti-tumor activity and reduce side effects.

## Data Availability

Not applicable.

## Abbreviations

5-HT: 5-Hydroxytryptamine
A2aR: A2a receptor
AKT: Protein kinase B
ALK: Anaplastic receptor tyrosine kinase
AMPK: Adenosine 5′-monophosphate-activated protein kinase
AP-1: Activator protein 1
AR: Androgen receptor
ARK: AXL receptor tyrosine kinase
BCAA: Branched chain amino acid
BCL-2: B-cell lymphoma-2
BMM: Bone marrow mononuclear
BRAF: B-Raf proto-oncogene
CCSC: Colon cancer stem cells
CD: Cluster of differentiation
CHK1: Checkpoint kinase 1
CIP2A: Cancerous inhibitor of protein phosphatase 2A
DC: Dendritic cell
DDR1: Discoid domain receptor 1
DNA-PK: DNA dependent protein kinase
E2F1: E2F transcription factor 1
EGFR: Epidermal growth factor receptor
ERα: Estrogen receptor α
ERK: Extracellular regulated protein kinases
FA: Fatty acid
FASN: Fatty acid synthase
FFAs: Free fatty acids
FRA1: FOS related antigen-1
GC: Gastric cancer
GSK-3: Glycogen synthase kinase-3
H2AX: H2A histone family member X
HBV: Hepatitis B virus
HCC: Hepatic cell carcinoma
HIF-1α: Hypoxia-inducible factor-1α
HNSCC: Head and neck squamous cell carcinoma
HSP: Heat shock protein
IKK: Inhibitor of nuclear factor kappa-B kinase
IL: Interleukin
IL-6R: Interleukin-6 receptor
IRS: Insulin receptor substrate
KRAS: Kirsten rat sarcoma viral oncogene
LCNEC: Large-cell neuroendocrine carcinoma
LKB1: Liver kinase B1
lncRNA: Long non-coding RNA
MAPKAP1: Mitogen-activated protein kinase associated protein 1
MEK: MAPK/ERK kinase
miRNA: microRNA
MMP-9: Matrix metallopeptidase 9
mTOR: Mammalian target of rapamycin
mTORC1: Mammalian target of rapamycin complex 1
mTORC2: Mammalian target of rapamycin complex 2
NF-κB: Nuclear factor kappa-B
NK: Natural killer
NMSC: Nonmelanoma skin cancer
NSCLC: Non-small cell lung cancer
OCCC: Ovarian clear cell carcinoma
OSCC: Oral squamous cell carcinoma
OTUD7B: OTU deubiquitinase 7B
PAAD: Pancreatic adenocarcinoma
PCNSL: Primary central nervous system lymphoma
PI3K: Phosphoinositide-3-kinase
PIK3R1: Phosphoinositide-3-kinase regulatory subunit 1
PIP2: Phosphatidylinositol (4,5)-bisphosphate
PIP3: Phosphatidylinositol (3,4,5)-trisphosphate
PK: Pyruvate kinase
PKM2: Pyruvate kinase M2
pNET: Pancreatic neuroendocrine tumors
PP2A: Protein phosphatase 2A
PTEN: Phosphate and tension homology deleted on chromosome ten
RCC: Renal cell carcinoma
RNF168: Ring finger protein 168
ROS: Reactive oxygen species
SCLC: Small cell lung cancer
SEGA: Subependymal giants-cell astrocytoma
SLC7A5: Solute carrier family 7 member 5
SREBP: Sterol regulatory element binding protein
STAT5: Signal transducer and activator of transcription 5
TFE: Transcription factor enhancer
TKI: Tyrosine kinase inhibitors
TM4SF1: Transmembrane-4 L-six family member-1
TRAF2: TNF receptor associated factor 2
TSC1: Tuberous sclerosis complex subunit 1
TSC2: Tuberous sclerosis complex subunit 2
UCP2: Uncoupling protein 2
VEGF: Vascular endothelial growth factor
